# Intermittent hypoxia produces Alzheimer disease?

**DOI:** 10.18632/oncotarget.18214

**Published:** 2017-05-26

**Authors:** Sosuke Yagishita, Akira Hirasawa

**Affiliations:** Department of Peripheral Nervous System Research, National Institute of Neuroscience, National Center of Neurology and Psychiatry, Tokyo, Japan

**Keywords:** intermittent hypoxia, sleep-disordered breathing, Alzheimer disease, tau, Gene Ontology

Alzheimer disease (AD) is the most common type of dementia, and the neuropathological hallmarks of AD are depositions of amyloid β protein (Aβ) (senile plaques) and hyperphosphorylated tau (neurofibrillary tangles). Accumulating evidence have indicated that aging is the major risk factor of AD, however, lack of suitable models has complicated studying the relationship between aging and AD. In our recent study [1], we have provide some evidence that intermittent hypoxia treatment (IHT) could be a novel tool compensating for this lack.

IHT is well known as an experimental model of sleep-disordered breathing (SDB). SDB is characterized by recurrent arousals from sleep and intermittent hypoxemia. Several clinical studies have suggested the close relationship between SDB and dementia including AD. For example, a recent meta analysis revealed that AD patients have a five times higher chance of presenting with obstructive sleep apnea (the most common form of SDB) than cognitively non-impaired individuals of similar age [2]. Thus we investigated whether IHT could be a model for dementia research.

IHT was performed as follows: mice were exposed to a protocol of 1 min of pure N_2_ injection, in order to reduce the fraction of inspired O_2_ (FIO_2_) from 21% to 5%, followed by 2 min of room air injection to increase FIO_2_ from 5% to 21%. This 3 min cycle was repeated for 8 h each day (from 9:00 am to 5:00 pm). We exposed mice to the protocol for 5 days or 28 days, and subjected their hippocampi for microarray analyses and biochemical analyses. Our major findings are as follows:

1. IHT and aging shared common biological processes.
2. IHT led to an increase in phosphorylated tau.

At first, we performed Gene Ontology (GO)-based microarray analyses that was previously established by Dr. Hirasawa and his colleagues [3]. We included data derived from mice reared for 12 months (referred to “aging”). Our experimental data were compared with the various other data published in Gene Expression Omnibus (The National Center for Biotechnology Information), and hierarchical clustering was performed to calculate the linkage distances among the experimental data. Interestingly, we revealed that IHT and aging shared alterations in some common GO terms, e.c. RNA metabolism, neurogenesis, cell cycle, energy production. It has been indicated that IHT caused aging processes [4], and our results may be the first experimental evidence for that. Our analyses also revealed that the following experimental data had similar comprehensive gene expression to IHT and aging: kainic acid treatment, Dicer ablation, or moderate glutamate excess. Kainic acid treatment and moderate glutamate excess led to neuronal excitation. Meanwhile, Dicer ablation led to an exaggerated seizure response. It is presumable that IHT could be closely related to neuronal excitation.

**Figure 1:**
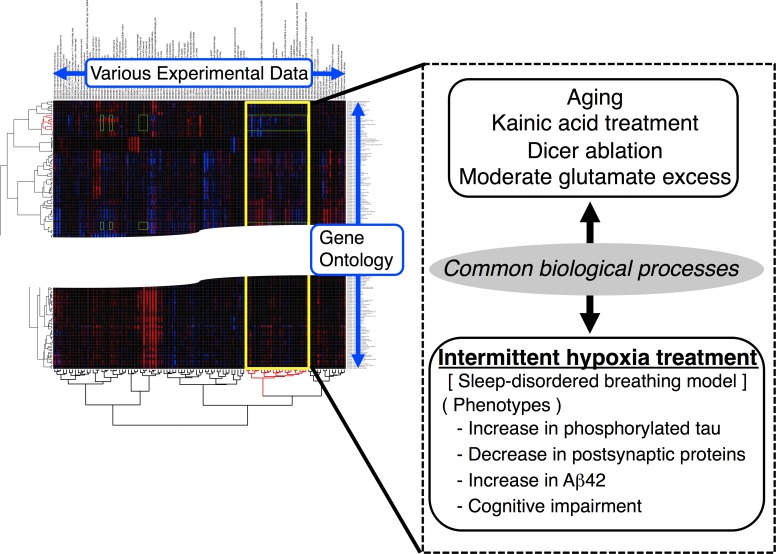
Intermittent hypoxia treatment could be a novel Alzheimer disease model

Next, we focused on tau phosphorylation because hyperphosphorylated tau accumulates in AD brains. Meanwhile, our *in silico* analyses suggested that IHT and aging cause alteration in balance of kinases and/or phosphatases, of which tau is a substrate. We revealed that IHT caused a significant increase in tau phosphorylation. This result was consistent with a previous report showing that Dicer ablation led to increase in phosphorylated tau [5], ensuring the validity of our GO-based microarray analyses. In parallel, IHT increased phosphorylated p70 S6 kinase, indicating involvement of the mTOR signaling pathway. Moreover, elongation of the IHT periods to 28 days resulted in decreases in postsynaptic proteins, suggesting a certain role of phosphorylated tau in regulating synaptic receptors [6].

In summary, IHT causes alterations in GO terms common to aging, a major risk factor for AD, and results in an increase in phosphorylated tau. It is a disputable question whether an increase in tau phosphorylation can be a risk factor for the AD onset, but we could say that IHT had the potential for providing a situation in which neurofibrillary tangle formation could occur. Meanwhile, according to a previous study, IHT causes an increase in the level of Aβ42, an Aβ species closely related to AD onset [7]. Moreover, the involvement of increased hippocampal activity and/or neuronal excitotoxicity in AD have been discussed. A recent study supports a model in which early development of occult hippocampal hyperexcitability may contribute to the pathogenesis of AD [8]. Given this model, it is considered reasonable that our microarray analyses noted the close relationship between IHT and neuronal excitation. These facts suggest that IHT may mimic an environmental stimulus that contributes to AD progression, and should be suitable for studying how and why one acquires susceptibility to AD with aging. Note that this model can be generated by just changing the rearing system of animals. It can be applied for various transgenic mice, rats, or marmosets.

Importantly, our microarray analyses noted the distinct gene expression patterns between our IHT model and a tau transgenic mouse model (rTg4510). At this moment, we interpreted the difference as meaning that the IHT model reflects the “presymptomatic” or “preclinical” stages; the rTg4510 model reflects the late stages after the onset.

Our findings are also important because of providing experimental evidence for understanding the potential relationship between SDB and AD. Cognitive impairments observed in SDB patients are associated with intermittent hypoxia, and rats or mice exposed to IHT demonstrated memory impairment. However, the mechanisms of IHT-induced cognitive impairments have not been elucidated. Several previous reports suggested that an increase in phosphorylated tau is closely relevant to cognitive impairments. Taken together with our findings, IHT-induced cognitive impairment may be partially attributed to the increase in phosphorylated tau.
